# Erratum to “Revealing patient-reported experiences in healthcare from social media using the design-acquire-process-model-analyse-visualise framework”

**DOI:** 10.1177/20552076241304176

**Published:** 2024-12-20

Murray C, Mitchell L, Tuke J and Mackay M. Revealing patient-reported experiences in healthcare from social media using the design-acquire-process-model-analyse-visualise framework. *Digital Health*. 2024;10. DOI:10.1177/20552076241251715.

In the above-mentioned article, the title has been corrected from “Revealing patient-reported experiences in healthcare from social media using thedesign-acquire-process-model-analyse-visualise framework” to “Revealing patient-reported experiences in healthcare from social media using the design-acquire-process-model-analyse-visualise framework”.

The article has been updated to reflect the correction.

