# Supplementary material for: Revealing patient-reported experiences in healthcare from social media using thedesign-acquire-process-model-analyse-visualise framework
Source: Digit Health. 2024 May 15;10:20552076241251715. doi: 10.1177/20552076241251715 (PMC11097732; doi:10.1177/20552076241251715)
Supplement: sj-pdf-1-dhj-10.1177_20552076241251715 - Supplemental material for Revealing patient-reported experiences in healthcare from social media using thedesign-acquire-process-model-analyse-visualise framework [file sj-pdf-1-dhj-10.1177_20552076241251715.pdf]

51. Murray CJ and Frenk J (2000) A framework for assessing the performance of health systems. *Bulletin of the World Health Organization* 78: 717–731.
52. Németh G (2006) Health related quality of life outcome instruments. *European Spine Journal* 15(1): S44–S51.
53. Nguyen DQ, Billingsley R, Du L and Johnson M (2015) Improving topic models with latent feature word representations. *Transactions of the Association for Computational Linguistics* 3: 299–313.
54. Nielsen FÅ (2011) A new anew: Evaluation of a word list for sentiment analysis in microblogs. *arXiv preprint arXiv:1103.2903* .
55. Okon E, Rachakonda V, Hong HJ, Callison-Burch C and Lipoff JB (2020) Natural language processing of Reddit data to evaluate dermatology patient experiences and therapeutics. *Journal of the American Academy of Dermatology* 83(3): 803–808.
56. Organization WH et al. (2017) *Technical series on safer primary care*. World Health Organization.
57. Pappa D and Stergioulas LK (2019) Harnessing social media data for pharmacovigilance: a review of current state of the art, challenges and future directions. *International Journal of Data Science and Analytics* 8(2): 113–135.
58. Peixoto TP (2014) Hierarchical block structures and high-resolution model selection in large networks. *Physical Review X* 4(1): 011047.
59. Peixoto TP (2017) Nonparametric Bayesian inference of the microcanonical stochastic block model. *Physical Review E* 95(1): 012317.
60. Piantadosi ST (2014) Zipf's word frequency law in natural language: A critical review and future directions. *Psychonomic bulletin & review* 21(5): 1112–1130.
61. Rainie L, Smith A, Schlozman KL, Brady H, Verba S et al. (2012) Social media and political engagement. *Pew Internet & American Life Project* 19(1): 2–13.
62. Reagan AJ, Mitchell L, Kiley D, Danforth CM and Dodds PS (2016) The emotional arcs of stories are dominated by six basic shapes. *EPJ Data Science* 5(1): 1–12.
63. Reddit I (2021) Reddit by the Numbers. URL https://www.redditinc.com/press. Accessed: 2022-06-29.
64. Schroepfer M (2018) An update on our plans to restrict data access on Facebook. *Facebook Newsroom* 4.
65. Silge J and Robinson D (2016) tidytext: Text mining and analysis using tidy data principles in r. *Journal of Open Source Software* 1(3): 37.
66. Silge J and Robinson D (2017) *Text mining with R: A tidy approach*. " O'Reilly Media, Inc.".
67. Tan AH et al. (1999) Text mining: The state of the art and the challenges. In: *Proceedings of the pakdd 1999 workshop on knowledge disocovery from advanced databases*, volume 8. pp. 65–70.
68. Tanwar M, Duggal R and Khatri SK (2015) Unravelling unstructured data: A wealth of information in big data. In: *2015 4th International Conference on Reliability, Infocom Technologies and Optimization (ICRITO)(Trends and Future Directions)*. IEEE, pp. 1–6.
69. The National Health Information and Performance Principal Comittee (2017) The Australian Health Performance Framework. URL https://www.aihw.gov.au/getmedia/1c95574c-ac07-4126-8b7c-31eb29d9b381/OOS318_Attachment-1.pdf.aspx. Accessed: 2022-06-29.
70. Twitter I (2022) Twitter Announces First Quarter 2022 Results. URL https://s22.q4cdn.com/826641620/files/doc_financials/ Accessed: 2022-06-29.
71. Van der Maaten L and Hinton G (2008) Visualizing data using t-SNE. *Journal of Machine Learning Research* 9(11).
72. Vicsek L (2010) Issues in the Analysis of Focus Groups: Generalisability, Quantifiability, Treatment of Context and Quotations. *Qualitative Report* 15(1): 122–141.
73. Walker S, Mercea D and Bastos M (2019) The disinformation landscape and the lockdown of social platforms. *Information, Communication & Society* 22(11): 1531–1543. DOI:10.1080/1369118X.2019.1648536. URL doi.org/10.1080/1369118X.2019.1648536.
74. Wang W, Feng Y and Dai W (2018) Topic analysis of online reviews for two competitive products using latent Dirichlet allocation. *Electronic Commerce Research and Applications* 29: 142–156. DOI: https://doi.org/10.1016/j.elerap.2018.04.003. URL https://www.sciencedirect.com/science/article/pii/S1
75. Weldring T and Smith SM (2013) Article Commentary: Patient-Reported Outcomes (PROs) and Patient-Reported Outcome Measures (PROMs). *Health Services Insights* 6: HSI.S11093. DOI:10.4137/HSI.S11093. URL https://doi.org/10.4137/HSI.S11093. PMID: 25114561.
76. Wickham H, Averick M, Bryan J, Chang W, McGowan LD, François R, Grolemund G, Hayes A, Henry L, Hester J et al. (2019) Welcome to the tidyverse. *Journal of open source software* 4(43): 1686.
77. Wold S, Esbensen K and Geladi P (1987) Principal component analysis. *Chemometrics and intelligent laboratory systems* 2(1-3): 37–52.
78. Zhou L, Zhang D, Yang CC and Wang Y (2018) Harnessing social media for health information management. *Electronic Commerce Research and Applications* 27: 139–151. DOI:https://doi.org/10.1016/j.elerap.2017.12.003. URL https://www.sciencedirect.com/science/article/pii/S1

Supplementary Materials

Table 4 shows the Australian Hospital Patient Experience Question Set. Table 3 shows the Google Scholar search terms from search keys seen in Table 1.

Table 3. Key for Table 1 showing the exact Google Scholar search term for each Search Key. The term `[social media platform]` is a placeholder for the social media platform names; Facebook, Reddit, and Twitter.

Search Key	Google Scholar Search Term
-	`[social media platform]`
Patient Experience	`[social media platform] AND 'patient experience' OR 'patient experiences' OR 'patient reported experience' OR 'patient reported experiences'`
Natural Language Processing	`[social media platform] AND 'natural language processing' OR 'nlp'`
Patient Experience + Natural Language Processing	`[social media platform] AND 'patient experience' OR 'patient experiences' OR 'patient reported experience' OR 'patient reported experiences' AND 'natural language processing' OR 'nlp'`

Table 4. Australian Hospital Patient Experience Question Set (AHPEQS), a core set of satisfaction questions that hospitals can use to capture patient-reported experiences[5].

Survey Questions	Response options
My views and concerns were listened to	Always; Mostly; Sometimes; Rarely; Never; Didn't apply
My individual needs were met (if answer always/mostly, skip to Q4)	Always; Mostly; Sometimes; Rarely; Never
When a need could not be met, staff explained why	Always; Mostly; Sometimes; Rarely; Never
I felt cared for	Always; Mostly; Sometimes; Rarely; Never
I was involved as much as I wanted in making decisions about my treatment and care	Always; Mostly; Sometimes; Rarely; Never
I was kept informed as much as I wanted about my treatment and care	Always; Mostly; Sometimes; Rarely; Never
As far as I could tell, the staff involved in my care communicated with each other about my treatment	Always; Mostly; Sometimes; Rarely; Never; Didn't apply
I received pain relief that met my needs	Always; Mostly; Sometimes; Rarely; Never; Didn't apply
When I was in the hospital, I felt confident in the safety of my treatment and care	Always; Mostly; Sometimes; Rarely; Never
I experienced unexpected harm or distress as a result of my treatment or care ([if answer is no, skip to Q12)	Yes, physical harm; Yes, emotional distress; Yes, both; No
My harm or distress was discussed with me by staff	Yes; No; Not sure; Didn't want to discuss it
Overall, the quality of the treatment and care I received was:	Very good; Good; Average; Poor; Very poor
